# Sensogenomics of music and Alzheimer’s disease: An interdisciplinary view from neuroscience, transcriptomics, and epigenomics

**DOI:** 10.3389/fnagi.2023.1063536

**Published:** 2023-02-03

**Authors:** Laura Navarro, Alberto Gómez-Carballa, Sara Pischedda, Julián Montoto-Louzao, Sandra Viz-Lasheras, Alba Camino-Mera, Thomas Hinault, Federico Martinón-Torres, Antonio Salas

**Affiliations:** ^1^Genetics, Vaccines and Infections Research Group (GENVIP), Instituto de Investigación Sanitaria de Santiago, Universidade de Santiago de Compostela, Santiago de Compostela, Galicia, Spain; ^2^Unidade de Xenética, Instituto de Ciencias Forenses, Facultade de Medicina, Universidade de Santiago de Compostela, and GenPoB Research Group, Instituto de Investigación Sanitaria (IDIS), Hospital Clínico Universitario de Santiago (SERGAS), Santiago de Compostela, Galicia, Spain; ^3^Centro de Investigación Biomédica en Red de Enfermedades Respiratorias (CIBER-ES), Madrid, Spain; ^4^Normandie Université, UNICAEN, PSL Université Paris, EPHE, Inserm, U1077, CHU de Caen, Centre Cyceron, Neuropsychologie et Imagerie de la Mémoire Humaine, Caen, France; ^5^Translational Pediatrics and Infectious Diseases, Department of Pediatrics, Hospital Clínico Universitario de Santiago de Compostela, Santiago de Compostela, Galicia, Spain

**Keywords:** Alzheimer’s disease, music stimuli, RNAseq, genes, dopamine, transcriptome, epigenome

## Abstract

**Introduction:**

The relationship between music and Alzheimer’s disease (AD) has been approached by different disciplines, but most of our outstanding comes from neuroscience.

**Methods:**

First, we systematically reviewed the state-of-the-art of neuroscience and cognitive sciences research on music and AD (>100 studies), and the progress made on the therapeutic impact of music stimuli in memory. Next, we meta-analyzed transcriptomic and epigenomic data of AD patients to search for commonalities with genes and pathways previously connected to music in genome association, epigenetic, and gene expression studies.

**Results:**

Our findings indicate that >93% of the neuroscience/ cognitive sciences studies indicate at least one beneficial effect of music on patients with neurodegenerative diseases, being improvements on memory and cognition the most frequent outcomes; other common benefits were on social behavior, mood and emotion, anxiety and agitation, quality of life, and depression. Out of the 334 music-related genes, 127 (38%) were found to be linked to epigenome/transcriptome analysis in AD (vs. healthy controls); some of them (*SNCA, SLC6A4, ASCC2, FTH1, PLAUR* and *ARHGAP26*) have been reported to be associated e.g. with musical aptitude and music effect on the transcriptome. Other music-related genes (*GMPR, SELENBP1* and *ADIPOR1*) associated to neuropsychiatric, neurodegenerative diseases and music performance, emerged as hub genes in consensus co-expression modules detected between AD and music estimulated transcriptomes. In addition, we found connections between music, AD and dopamine related genes, with SCNA being the most remarkable – a gene previously associated with learning and memory, and neurodegenerative disorders (e.g., Parkinson’s disease and AD).

**Discussion:**

The present study indicate that the vast majority of neuroscientific studies unambiguously show that music has a beneficial effect on health, being the most common benefits relevant to Alzheimer’s disease. These findings illuminate a new roadmap for genetic research in neurosciences, and musical interventions in AD and other neurodegenerative conditions.

## 1. Introduction

Over 55 million people live with dementia (Gauthier et al., 2021). Alzheimer’s disease (AD) is a well-known and the most common form of dementia, in which “brain cells and nerves are blocked by abnormal proteins, resulting in the disruption of the transmitters which carry messages in the brain, particularly those responsible for storing memories” (Gauthier et al., 2021). The large number of people affected worldwide, and the complexity of this neurodegenerative disease, constitute a significant challenge for science in general, and specifically for every discipline seeking to further understand this illness.

The relationship between music and AD has been approached from different perspectives in neuroscience and cognitive sciences, often aiming at understanding the mechanisms underlying human memory, e.g., (Groussard et al., 2019). Neuroscience has identified three main lines connecting music and AD. First, the identification of certain types of musical memory preserved in AD patients is one of the most promising areas for clinical research (Cuddy et al., 2015; Jacobsen et al., 2015). Second, musical training can induce brain structural changes, thereby engaging brain plasticity (Fauvel et al., 2013; Groussard et al., 2014). Third, a growing number of studies in psychophysiology have highlighted how music can positively modulate biological markers (e.g., Mockel et al., 1994; Kreutz et al., 2004; Etzel et al., 2006; Carpentier and Potter, 2007; Fancourt et al., 2014; Zatorre, 2015; Gauthier et al., 2021). Music has also been considered a powerful tool in rehabilitation programs (Sarkamo, 2018), but the biological mechanism underlying the therapeutical effects of music remains unclear.

Memory loss is the key symptom of AD. Its formation and maintenance have been strongly associated with epigenetic modifications, and DNA methylation induces dynamic and stable changes in the adult central nervous system (CNS) (Zovkic et al., 2013). The field of neuroepigenetics has recently emerged (Sweatt, 2013) with the aim to interrogate the specific role of epigenetic mechanisms in the regulation of the CNS in terms of acquired behaviors, neurological disorders, neural plasticity, etc. However, the relationship between epigenetics and music remains to be understood. According to the “environmental epigenetics” hypothesis (Brigati et al., 2011), it seems plausible that music could acts as an epigenetic modulator, able to regulate gene expression, with possible effects in brain plasticity. Thus, music might shape the brain by modifying the epigenome, and lead to sustained alterations in its structure (Brigati et al., 2011).

A few studies have also been carried out recently to explain the effect of musical stimuli on the transcriptomes, connecting music with memory and neurodegeneration. Kanduri et al. (2015b) analyzed the effects of gene expression after listening to classical music, and reported a few genes that could be relevant to research on AD. For instance, they detected upregulated genes related to learning, memory, cognitive performance, neuroprotection, neurogenesis and synaptic neurotransmission, and also a few downregulated genes responsible for neurodegeneration (e.g., neuronal apoptosis). The study by Nair et al. (2020) identified upregulation of six miRNAs related to neurodegeneration, dopamine metabolism, neuronal activity, modulators of neuronal plasticity, CNS myelination and cognitive functions, such as long-term potentiation and memory. Interestingly, among the best miRNA candidates, these authors found the miR-132, a miRNA that is known to regulate the TAU protein, which has been connected to AD prevention (Lantero-Rodriguez et al., 2021). The study carried out by Kanduri et al. (2015a) on professional musicians detected several over-expressed genes, some of them related to dopaminergic transmission and neurocognitive functions, such as learning and memory. A few years later, Nair et al. (2019) analyzed gene expression of miRNAs after music performance; among other findings, they reported two up-regulated miRNAs that target *FOXP2* and constitute a miRNA-*FOXP2* gene regulatory network, in which some of the molecules were important long-term potentiation (LTP) and dopamine signaling members. These authors also reported up-regulated miRNA related to memory formation, motor neuron functions and neural plasticity. Finally, the connection between music and dopamine has also been explored from different perspectives, including cognitive sciences and neurosciences (Wise, 2004; Menon and Levitin, 2005; Salimpoor et al., 2013; Strange et al., 2014; Zatorre, 2015; Ji et al., 2016; Lewis et al., 2019), and transcriptomics (Emanuele et al., 2010; Järvelä, 2018).

Recent population-based genetic association studies have also been carried out to explore the connections between music and memory, and a few candidate genes were identified. Järvelä (2018) highlights a few genes connecting music with memory in animals, e.g., *PCDHA1-9* gene related to memory in mice (Hertel et al., 2012; Lin et al., 2012), *GRIN2B* related to brain plasticity (Pfenning et al., 2014), or *EGR1* related to reward-related synaptic plasticity (Avey et al., 2008; Drnevich et al., 2012). Some genes have also been found to be statistically associated with musical memory in humans. For instance, molecular genetic studies in human behavior have highlighted the role of *AVPR1* and *OXTR* in connection with musical abilities such as musical memory (Israel et al., 2008). *AVPR1A* has also been associated with musicality (Mariath et al., 2017), musical memory (Granot et al., 2007, 2013), memory and learning (Fink et al., 2007). Other genes of interest would be: *SLC6A4* [associated to musical memory (Granot et al., 2007, 2013)], *KCTD8* (Metz et al., 2011), and *PCDHA1-9* (Ukkola-Vuoti et al., 2013).

Navarro et al. (2021) have recently reviewed the genetic background of several musical phenotypes and conditions. This study highlighted the interest of analyzing the impact of music stimuli on gene expression, as part of a new discipline called ‘sensogenomics’ (https://sensogenomics.com). Sensogenomics represents a call for more intense research on genomics, on the basis of emerging and convergent evidence that points to a real genetic impact of music as a positive reward stimulus in AD patients.

Against this background, the aim of the present study is twofold: first, systematically review previous research investigating music as a stimulus for AD patients, focusing on the advances made in neurosciences and cognitive sciences as the area that has contributed more profusely to this field and focusing on the impact of music on health. Secondly, in line with our previous conceptualization of sensogenomics (Navarro et al., 2021), and to overcome the scarcity of studies on music and AD, here we develop a novel ‘omic’ approach aimed at disentangling commonalities between (i) genes that are altered in AD patients (inferred from transcriptomic and epigenomic studies available in the public domain), and (ii) genes that have been shown to be associated with different conditions related to music. Although causal relationships for this commonality cannot be ascertained with the available data, these links might illuminate new frontiers for neurological research and musical interventions in AD and other neurodegenerative conditions.

## 2. Methodology

### 2.1. Literature search

**Figure 1:**
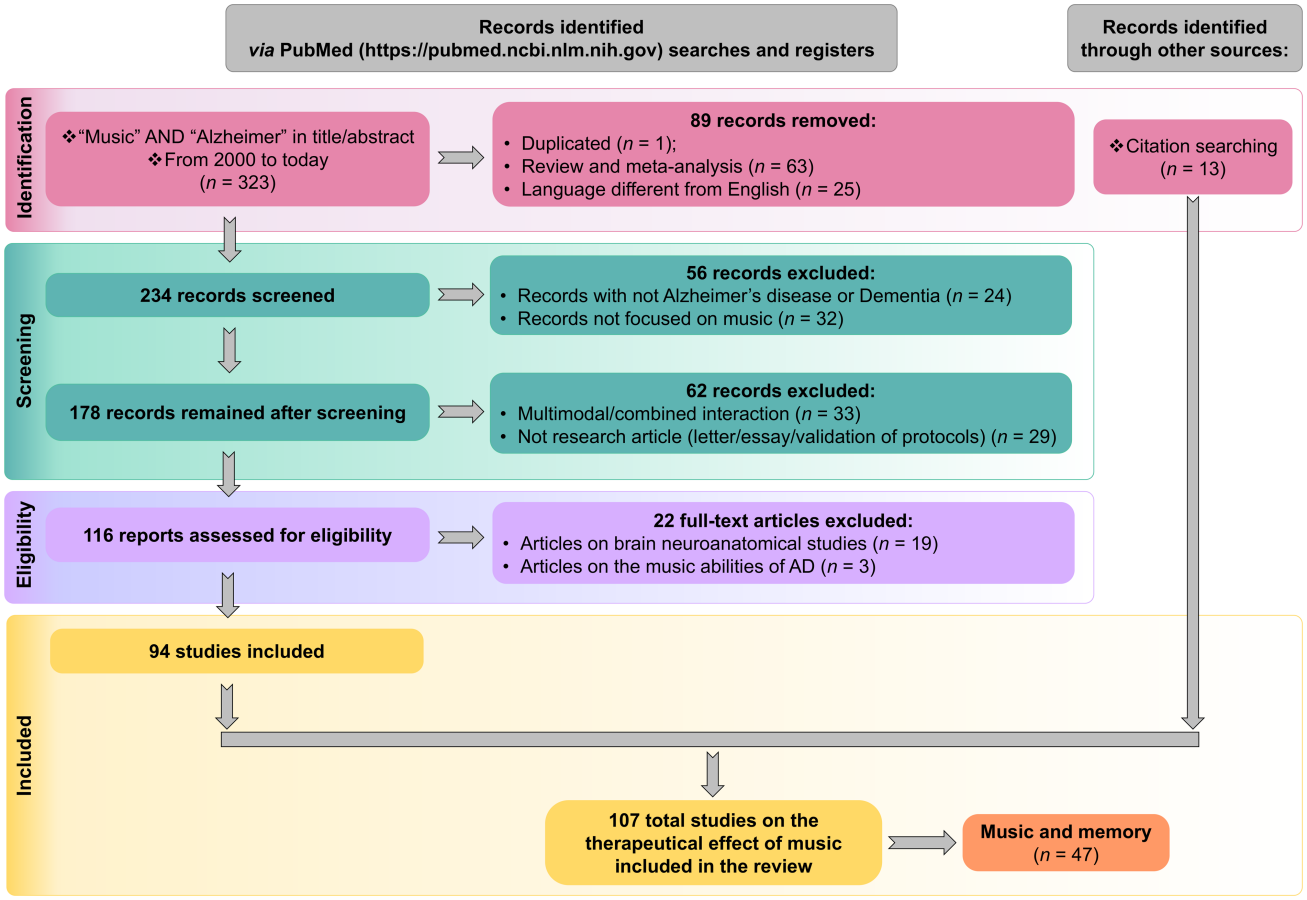
The systematical review adhered to PRISMA guidelines and following the flow diagram described in the scheme.

**Figure 2:**
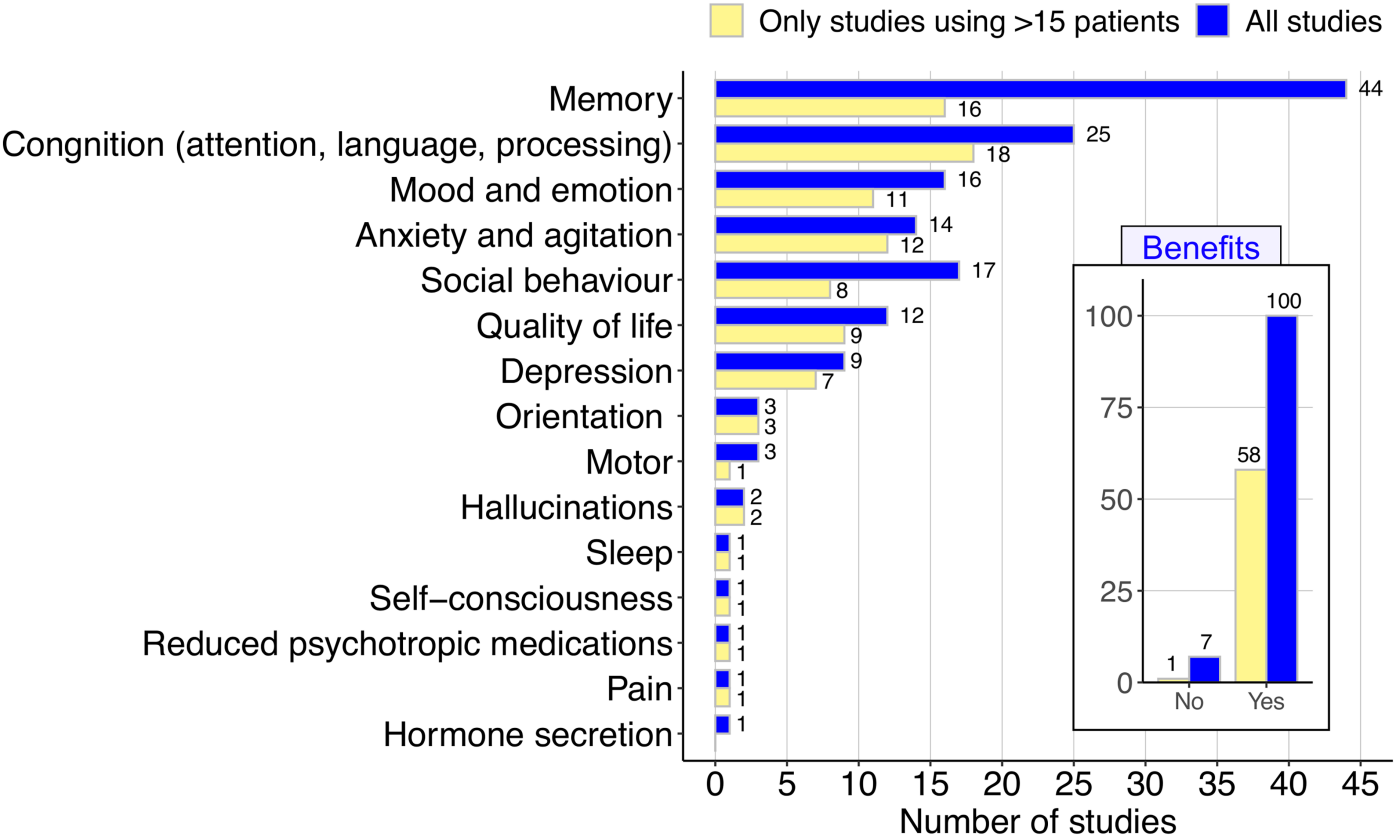
Summary of the beneficial effects of music in AD as reported in a total of 107 articles (see Figure 1). The inset figure indicates the number of articles that report some benefit vs. those with negative findings for the benefits; while the main figure describes with more detail the different benefits reported. The graphic was carried out considering the total amount of articles (blue), and only those based on the analysis of a minimum of patients (>15).

The systematic review of articles related to music and AD was carried out according to the Preferred Reporting Items for Systematic Reviews and Meta-analyses (PRISMA) guidelines. Indexed searches were performed in PubMed using the following query “music” AND “Alzheimer,” in title and abstract. The search yielded 323 papers; 217 were excluded after close inspection following the sequential criteria indicated in the PRISMA scheme (Figure 1; Supplementary Table S1); the reasons for exclusions included: duplicated articles, reviews and meta-analysis, articles not focused on AD or music and articles written in a non-English language, among others. We also disregarded a few articles dealing with neuroanatomical studies or music abilities of AD, because the focus of the present review was placed on the benefits and therapeutic effects of music (Figure 2). Of the 107 articles dealing with the therapeutical effect of music, a subset of them were related to music and memory (*n* = 47), an outcome particularly relevant to AD.

Moreover, a total of 13 additional relevant articles were added to the list by scrutinizing references list of the selected articles and reviews (Figure 1).

Because our findings on the ‘omic’ side include a connection between gene expression and dopamine, we also carried out a PubMed search for the query “music” AND “dopamine*” to investigate the state-of-the-art in this terrain. This search returned 63 papers, of which only 12 of them were retained after close inspection for relevance (see Supplementary Text to check the most relevant information regarding this search).

### 2.2. Music-related genes

A total of 334 genes (Supplementary Table S2; Supplementary Figures S1, S2) were selected as candidates to be associated with musical traits. In a first approach, genes from the two main reviews on the matter were selected: Järvelä (2018) proposes a selection of top candidate genes for musical aptitude and music performance, while Navarro et al. (2021) carried out an exhaustive search in the literature related to music (mainly genetic association studies), and included genes that might be related to the impact of music on gene expression. This list was completed by revisiting the original papers studying the effect of music listening and music performance on gene expression (Kanduri et al., 2015a,b; Nair et al., 2019, 2020); and also other studies that, from wider perspectives, also aimed at connecting genes and musical traits. These include: (i) genes that fall in the top 20 regions identified by Liu et al. (2016) under an *F*_ST_ scrutiny method of the genome to find signatures of positive selection associated with musical aptitude; (ii) the top genes from Oikkonen’s study (Oikkonen et al., 2016) where convergent evidence for the molecular basis of musical traits was obtained through integration of gene-level data from 105 published studies; and (iii) genes related to the terms “music” or “musical” with relevant publications using *geneshots*;^1^ the query “music*” was performed under “GENERIF” (manually collected gene-term association). From the results of those queries, and after careful manual checking, only those with relevant publications were retained (for instance those related to human studies, candidate genes, etc.)

### 2.3. Epigenomic datasets and data analyses

The association between aberrant epigenetic modifications leading to dysregulation of gene expression and AD progression has been thoroughly investigated (Fenoglio et al., 2018; Stoccoro and Coppede, 2018; Nikolac Perkovic et al., 2021). We analyzed the panel of candidate genes related to music in connection to epigenomics in AD. We used the datasets from Nabais et al. (2021) (Gene Expression Omnibus [GEO]: GSE153712), which contain epigenetic data generated for the Illumina Infinium Human MethylationEPIC Beadchip (Illumina Inc., San Diego, CA) on 161 AD patients (91 females and 70 males), and 471 healthy individuals (272 females and 199 males).

First, the methylation values associated with each of the probes on the MethylationEPIC microarray were converted to *Beta* values, by calculating the ratio of methylated probe intensity over total intensity (methylated and unmethylated) for each probe. Samples and probes’ quality control was performed with the package *minfi*, which provides a quality control report on the basis of intrinsic control probes present in the array, in addition to allowing to remove probes and samples according to their signal intensity. Subsequently, to reduce the risk of measurement biases, raw Intensity Data files (*.idat) were filtered using *RnBeads* package. As usual, cross-reactive probes, probes located within three base pairs of common SNPs, probes with missing values or no variability in methylation, and those located on sex chromosomes, were removed. In the preprocessing step, *RnBeads* was also used to estimate sample donors’ sex based on their DNA methylation status – an important step to identify possible discrepancies between documented gender and biological sex. Background adjustment of the methylated and unmethylated intensities was performed using the Dasen method (Pidsley et al., 2013), while normalization of *Beta* values was carried out using the *BMIQ* normalization method (Teschendorff et al., 2013). To identify differentially methylated positions (DMPs), we used the *limma* package (Ritchie et al., 2015), which performs a linear model, adjusted for sex, to compare DNA methylation patterns between patients with AD and healthy controls. *DMRcate* package (Peters et al., 2015) was used to identify significantly differentially or variable methylated regions (DMRs). *methylGSA* package (Ren and Kuan, 2019) was employed to carry out the gene set testing and pathways analysis of genes associated with DMPs. This method faces the biggest challenge in performing gene set analysis, that is, assigning differentially methylated features to genes, and adjusting for the number of CpGs instead of gene length. For significant DMRs, we performed gene ontology (GO) and Kyoto Encyclopedia of Genes and Genomes (KEGG) pathway enrichment analysis, applying the functions *enrichGO* and *enrichKEGG* of the *ClusterProfiler* package (Wu et al., 2021).

*P*-values were corrected for multiple testing using the false discovery rate (FDR) method. Only DMPs with an FDR-adjusted *p*-value <0.05 were considered. DMRs were considered if having a minimum of three CpGs sites inside, and an FDR-adjusted *p*-value <0.05. The same thresholds were used also for gene-set and pathways analysis.

All the statistical analyses were carried out using R software (v.4.1.2).

### 2.4. Transcriptomic datasets and data analyses

We downloaded gene expression data from four independent microarray datasets located at the GEO database. Overall, these datasets include 972 blood samples from AD patients and healthy controls (HC): GSE140829 (*n* = 453; cases = 204, controls = 249), GSE63061 (*n* = 273; cases = 139, controls = 134), GSE63060 (*n* = 246; cases = 142, controls = 104) and GSE97760 (*n* = 19; cases = 9, controls = 10). The GSE97760 study was finally excluded from the meta-analysis due to: (i) the low number of samples available, and (ii) the fact that it was the only dataset coming from a different array platform (Agilent), reducing the number of common genes with the other datasets (all of them from Illumina Beadchip arrays v.3 and v.4).

For raw data processing, we first performed a normal-exponential background correction following a quantile normalization (after a Log_2_-transformation) of the raw data using *limma* package (Ritchie et al., 2015).

After data normalization, expression data captured by multiple probes belonging to the same gene were averaged. Because of the difference in background measurements for each dataset we used Combat CONormalization Using conTrols (COCONUT) (Sweeney et al., 2016) to correct for batch effects between experiments, and to make the data comparable. COCONUT is an unbiased co-normalization method that assumes that all HC across studies come from the same statistical distribution, estimating first correction factors from each dataset’s HC samples, and then applying them to the AD samples in each dataset. This procedure removes technical differences while still retaining within-dataset differences between HC and AD groups. To correct for internal batches in the GSE140829 dataset, we used the function *Removebatcheffect* from the *limma* package before COCONUT co-normalization.

Differential expression (DE) analysis between AD samples and HC was carried out with *limma* and using gender as covariate to correct the model. Volcano plots of differentially expressed genes (DEGs) were built with *EnhancedVolcano* (Blighe et al., 2020) and *Upsetplots* from DEGs, and music and dopamine related genes were generated with the *ComplexUpset* R package (Lex et al., 2014). We used the R package *Enrichmentbrowser* (Geistlinger et al., 2016) to collect genes involved in dopamine-related biological processes from GO database and searching for terms including “dopamine” (Supplementary Table S3). Over-representation analysis from music-related genes through GO biological processes was conducted using the *Clusterprofiler* (Wu et al., 2021) R package. We applied the Benjamini-Hochberg procedure for multiple test correction and thresholds were set to 0.05. Fold enrichment was calculated as the quotient from gene ratio (number of genes of interest which are annotated to the gene-set/total number of genes of interest) and background ratio (size of the gene-set/size of all the unique genes annotated in the reference database).

**Figure 3:**
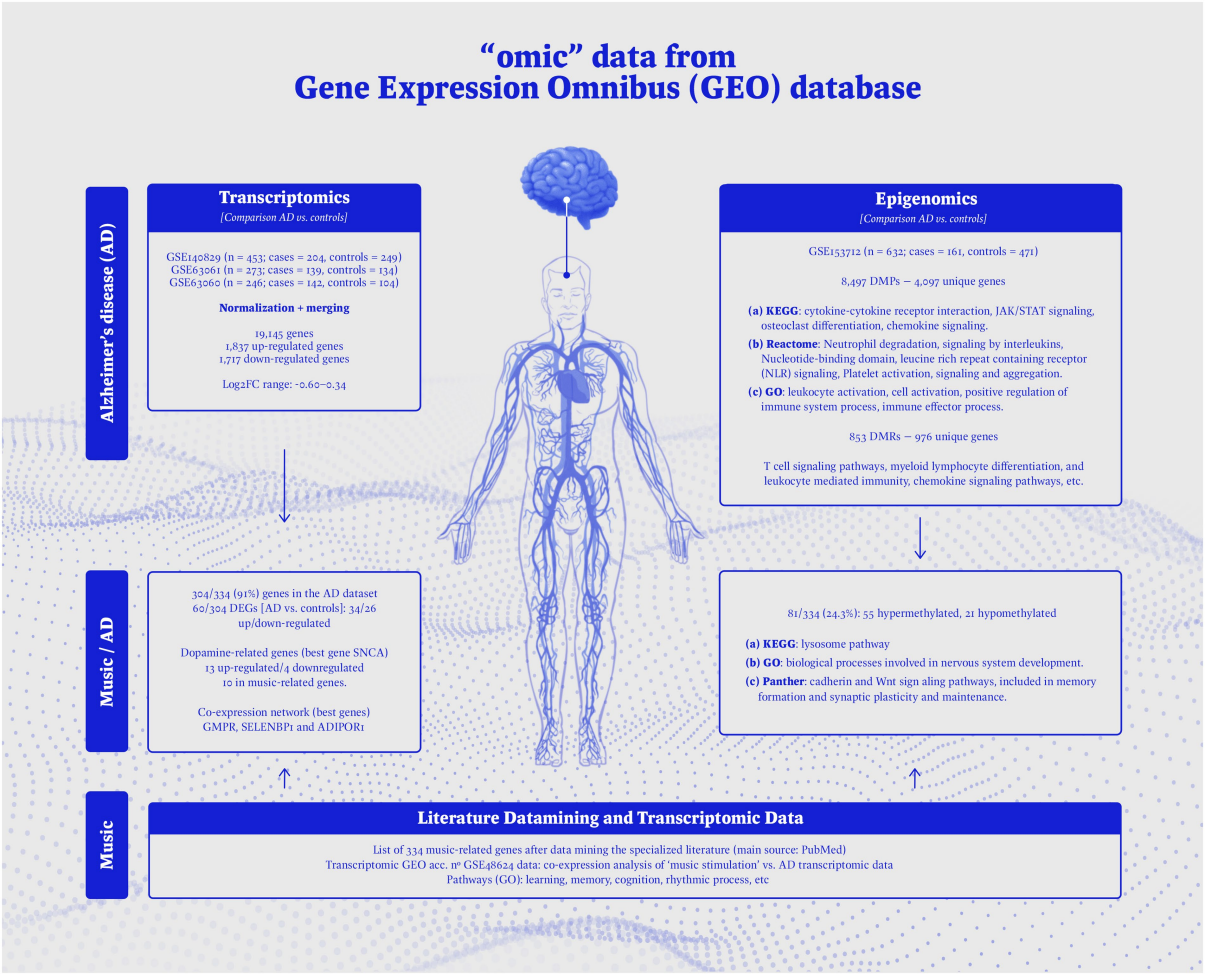
Overview on the -omic data used in the present study, and schematic representation of the main results obtained.

The flow diagram of Figure 3 provides an overview on the epigenomic, and transcriptomic data used in the present study, together with a schematic representation of the main findings.

### 2.5. Co-expression networks

The consensus modules from the global co-expression networks represent biologically robust co-expressed gene groups. The analysis carried out on the AD (vs. HC) and the music transcriptomic data reveal commonalities between both datasets, elucidating common coordinated genetic processes behind musical stimulation and AD. We studied the commonalities in network organization of gene expression between the AD dataset and a dataset including whole blood gene expression data from individuals before and after 20 min of classical music stimulation (GSE48624). For this purpose, we used only AD patients from the AD dataset, and samples collected after stimulation from the music dataset.

The consensus weighted gene co-expression network was constructed using the WGCNA R package (Langfelder and Horvath, 2008). We used as input normalized expression data (and corrected for differences in gender) from common genes (those represented in both datasets) that showed the most variant expression values between samples (top 75% with the highest variance). Then, independent datasets were integrated into a multi-set format suitable for consensus analysis. We followed the signed network procedure, whereby the similarity between genes reflects the sign of the correlation of their expression profiles. A matrix of correlations between all pairs of selected genes was generated from the expression values, and further converted into an adjacency matrix with a power function. We chose a soft-thresholding power based on the criterion of scale-free topology after testing a set of candidate powers. Considering that the model-fitting index of a perfect scale-free network is 1, we selected a soft-thresholding power of 5 (Supplementary Figure S3A) because it resulted in the maximum model fitting index for both datasets (>0.9). Subsequently, the consensus topological overlap matrix (TOM) from the adjacency matrices and the corresponding dissimilarity (1–TOM) values were computed. Considering the different properties of the datasets, we scaled TOMs to make them comparable (Supplementary Figure S3B). The consensus TOM was calculated with component-wise (‘parallel’) minimum of the TOMs for each set. As co-expression module detection parameters, we chose a minimum module size of 30, a medium sensitivity for cluster splitting, and a 0.2 as dendrogram cut heigh threshold for module merging. The resulting consensus modules or groups of co-expressed genes were labelled by colors and used to calculate module eigengenes (the first principal component of the module). Module membership (MM) was calculated as a measure of intramodular connectivity. The core genes within the most relevant modules were selected using a MM > 0.8.

## 3. Music as a powerful stimulus in Alzheimer’s disease

A recent review by Zhu et al. (2019) emphasizes the power of musical experience to activate the brain and postpone dementia, specially in AD. However, while the mechanism underlying the neurological processes remains unknown (see Supplementary Text: Neural Correlates of music in AD), numerous neuroscientific and psychological studies have tried to explain the function of music in different types of memories, and the effects of music in AD patients.

### 3.1. Music as a therapy in Alzheimer’s disease

There is a growing interest in cognitive sciences to explore the beneficial impact of music on AD and other neurodegenerative diseases (Arab et al., 2021; Li et al., 2022). Several fields of research have aimed at analyzing the effects of music in dementia (case studies, randomized clinical trial, quasi-experimental studies…); the number of available studies is high although the methodology employed very heterogeneous. In addition, there are also many ongoing pilot trials and study protocols aiming at evaluating the effectiveness of music in AD patients (Guétin et al., 2009; Belleville et al., 2019; Gulliver et al., 2019; Flo et al., 2022). Recent reviews (e.g., Leggieri et al., 2019) agree that participating in music activities improves behavioral and psychological symptoms.

Our systematic review of the relevant literature unequivocally demonstrates the beneficial effect of music in AD (Figure 2); there is a convergent and vast evidence emerging from the literature indicating an overall beneficial effect of music on rehabilitation and improvement of AD. Only 7 out of the 107 (6.5%) studies that survive the filters of our PRISMA selected criteria did not report a benefit of music in AD. The remaining studies (*n* = 100; 93.5%) all show some benefit of music, being the main outcome on the enhancing of memory (Figure 2), which represents the main disability of AD.

Memory is a complex cognitive activity, and different types of long-term memory (explicit and implicit) have been studied in regard to dementia (see Section 3.2). A pioneer clinical trial by Arroyo-Anlló et al. (2013) demonstrated that familiar music enhances self-consciousness and awareness, one of the main concerns in AD. Many other studies have highlighted music as a memory enhancer (Simmons-Stern et al., 2010), in which patients with AD demonstrated better recognition accuracy thanks to music mediation. Särkämö et al. (2014) showed that music listening improved remote episodic memory, mood and orientation. Gómez-Gallego and Gómez-Garcia (2017) demonstrated that music therapy improved psychological, social, and cognitive behaviors. Twenty six reviewed studies exposed the improvement of cognition after musical intervention, [see for example (Ceccato et al., 2012; Innes et al., 2018; Lyu et al., 2018)], general cognition and executive function (Doi et al., 2017; Innes et al., 2017; Kim et al., 2022), visuospatial processing (Maguire et al., 2015), language fluency and autobiographical narrations (Thompson et al., 2005; El Haj et al., 2013; Pongan et al., 2017).

There are many other studies focusing on the beneficial effects of music on the psychological or emotional states, specially reducing agitation and anxiety in persons with dementia (Svansdottir and Snaedal, 2006; Raglio et al., 2008; Cooke et al., 2010; Sung et al., 2012; Vink et al., 2013; Cohen-Mansfield, 2014; Narme et al., 2014; Gómez-Romero et al., 2017; Pedersen et al., 2017; Harrison et al., 2021). As a matter of fact, several authors have reported the positive impact of music on wellbeing and reduction of depression in persons with dementia (Guétin et al., 2009; Janata, 2012; de la Rubia Ortí et al., 2018; Ray and Gotell, 2018); while other studies reported the impact of music regarding the reduction of pain and the improvement of quality of life (Pongan et al., 2017). From a psycho-social perspective, music also enhances motivation and reward circuits in AD patients (Simmons-Stern et al., 2010), and stimulates social behavior. Group music participation provides support to caregivers and AD, stimulating meaningful interaction between them (Tamplin et al., 2018).

Also, some investigations aimed at studying the possibilities and limitations of different musical interventions, contrasting listening activities vs. more active musical intervention. Sakamoto et al. (2013) suggested that musical interactive interventions exhibited stronger beneficial emotional effect than music listening in individuals with severe dementia. Recent studies corroborate the large effect of music active intervention in cognition, behavior, and functional state in AD (Gómez-Gallego and Gómez-Garcia, 2017), and some of them based on singing or choral showed relevant results in AD patients (Pongan et al., 2017; Lyu et al., 2018; Pongan et al., 2020; Flo et al., 2022; McDowell et al., 2022).

The benefits of individualized music or person-centered music interventions in AD is a recurrent finding (Gerdner, 2000; El Haj et al., 2015; Ihara et al., 2019), being familiar music a powerful tool with AD patients. Overall, these studies demonstrate that music has the power to enhance the recall of their past personal history more than other activities (Lord and Garner, 1993). Clements-Cortes et al. (2016), in the context of music therapy, studied the potential of 40 Hz sensory brain stimulation in AD and Parkinson’s Disease patients. More than 100 reviewed studies as well as the most recent reviews (Leggieri et al., 2019; Matziorinis and Koelsch, 2022) highlight the beneficial effect of music therapy for AD management and the slowdown of neurodegeneration (Matziorinis and Koelsch, 2022). In the same line, a recent meta-analysis (Lai et al., 2020) proposes music therapy as the best treatment for Mild Cognitive Impairment (MCI).

### 3.2. Music, memory and Alzheimer’s disease

Music constitutes a distinct domain of non-verbal knowledge but shares certain cognitive organizational features with other brain knowledge systems (Omar et al., 2010). In line with a recent review (Baird and Samson, 2015), it should be considered that various forms of musical memory exists, and they may be differentially impaired in AD. In a systematic search aimed at analyzing the main findings related to different type of memories in studies focused on musical intervention in AD patients, we found 34 relevant articles (Figure 1). Main findings from this review are summarized in Table 1.

**Table 1:** Effects of music on different types of memory as recorded in the literature.

ID	Findings/Therapeutical effects of music in memory of AD patients	Type
40	Music boosts AM and the sense of identity	AM
111	Learning a favourite song improves AM recall and other cognitive abilities	AM
144	Music evokes AM	AM
199	Music enhances AM, specially own-chosen music.	AM
234	Popular songs have the ability to reminiscence AM	AM
240	Emotional and sad music would be the best to recall autobiographic experiences	AM
246	Music evokes AM	AM
290	Music boosts AM and reduces anxiety	AM
336	Music boosts AM and verbal expression	AM
97	Musical mnemonics may help people with AD learn verbal information that relates to their daily life	EM
189	Musical association enhances verbal EM	EM
237	Music as mnemonics strategy to retention	EM
331	Impaired musical memory in AD patients	EM
301	Musical recognition of familiar music is preserved + musical AM is preserved	EM + SM
329	musical recognition of familiar music is preserved + musical SM is preserved	EM + SM
209	Music aids mnemonics (learning sung lyrics aids retention)	EM + SM
226	Musical SM is preserved (remembering melodic lines and musical excerpts)	SM
213	No significant correlation between key and the attribution of ‘happy’ or ‘sad’ judgements to a musical piece could be found in all groups	SM
265	Recognition of musical instruments and emotion, and impaired recognition of compositions and musical symbols	SM
274	Musical emotional judgment remains intact in AD	SM
330	Semantic memory for melody may be preserved	SM
333	Musical semantic memory is preserved	SM
201	Semantic mus memory is preserved, music stimulates memory, preservation of musical PM	SM + PM
332	Intact mere exposure effect	IM + EM
113	IM remains intact: preference for familiar stimuli (mere exposure effect)	IM
163	Preservation musical abilities and memory: learn a new song, intact IM function	IM
164	Music modulates memory of songs	IM
196	AD patients score similarly to controls in the musical imagery tasks	IM
269	Preserved musical memory (IM), music can modulate memory functions (strong emotional power)	IM
319	Intact mere exposure effect for healthy older adults but not for patients with AD	IM
195	Preservation of musical PM	PM
334	Preservation of PM to play an instrument	PM
335	Preservation of PM to play an instrument	PM
210	Music aids mnemonics (retention) to learn motor and gestures	MM

The relevant articles were searched following the PRISMA flow diagram of Figure 1. See Supplementary Table S1 for References IDs. AM, autobiographical memory; EM, episodic memory; SM, semantic memory; PM, procedural memory; IM, implicit memory; MM, motor memory.

Nine articles studied autobiographical memory, considered to be of a mainly episodic nature (related to personal past events), and that is generally deteriorated in AD patients. Clinical trials, reports pre-post intervention, and case studies, have demonstrated the power of music to stimulate autobiographical memory (Irish et al., 2006; El Haj et al., 2012). Music constitutes a powerful stimulus that retrieves autobiographical, involuntary, and spontaneous memories in AD patients (Irish et al., 2006; El Haj et al., 2012, 2013, 2015; Fraile et al., 2019), reinforcing the sense of identity (Platel et al., 2021). Autobiographical memory has been considered an island of preservation during the progression of AD (Baird et al., 2018), while other authors have shown the power of popular songs (Basaglia-Pappas et al., 2013) or favorite songs (Fraile et al., 2019) to improve this kind of memory.

Some studies focused on how familiar music has been relatively spared by AD, and the patients’ intact recognition of familiar music (Vanstone et al., 2009). Other studies based on unfamiliar music found impaired episodic musical memory in AD patients (Quoniam et al., 2003; Menard and Belleville, 2009; Vanstone et al., 2012). Also, music is being used as a mnemonic strategy to enhance verbal episodic memory (Simmons-Stern et al., 2012; Moussard et al., 2014; Palisson et al., 2015; Ratovohery et al., 2019).

Semantic memory is a long-term memory related to more general knowledge and facts (grammar, name of colors, etc). In the context of music memory, it has been defined as “the information accessed by sense of familiarity for a melodic progression, regardless of timbre or starting pitch, and stripped from any contextual information” (Groussard et al., 2019); and also, as the ability to distinguish distorted melodies. Several studies have shown that musical semantic memory is highly preserved in AD (Cuddy and Duffin, 2005; Gagnon et al., 2009; Vanstone et al., 2009; Vanstone and Cuddy, 2010; Kerer et al., 2013; Cuddy et al., 2015). Kerer et al. (2013) showed that MCI and AD patients were impaired in tasks requiring verbal memory for music (recalling composers name, titles…) but, they performed better than healthy subjects at discriminating musical excerpts or remembering melodic lines. Omar et al. (2010) reported that AD patients recognized musical emotions and musical instruments but showed impaired recognition of compositions and musical symbols.

Many studies agreed in that implicit musical memory is well-preserved in AD patients (Baird et al., 2017; Deason et al., 2019), and specially procedural memory (Jacobsen et al., 2015). According to Groussard et al. (2019) “musical procedural memory is the ability to perform a previously learned musical motor sequence in a fluid manner.” Some pioneering case studies have focused on exploring the preservation of musical procedural memory to play an instrument (Beatty et al., 1988, 1994, 1997, 1999; Crystal et al., 1989; Cowles et al., 2003; Fornazzari et al., 2006). Also, other studies have reported the preservation of musical procedural memory in advanced AD (Cuddy et al., 2015; Jacobsen et al., 2015). Jacobsen et al. (2015) stated that “…[long term] musical [procedural] memory is surprisingly robust,” because the two brain regions that predetermine long-term memory encoding musical memory (caudal anterior cingulate gyrus and ventral supplementary motor area) have been preserved in advanced AD patients compared with other brain regions.

## 4. Music and Alzheimer’s disease: Insights from transcriptomics and epigenomics

### 4.1. Music-related genes

A total of 334 genes were selected through a thorough scrutiny of the literature and datasets (Supplementary Table S2). A narrow selection of 127 candidate genes was highlighted based on transcriptomic/epigenomic analyses (see below). Six of those were particularly prioritized as candidate genes, namely, *SNCA*, *SLC6A4*, *ASCC2*, *FTH1*, *PLAUR*, and *ARHGAP26*, because: (i) they were repeatedly found to be associated with musical aptitude in previous work and have emerged in transcription-based studies, and (ii) they occur consistently in the literature on AD.

**Figure 4:**
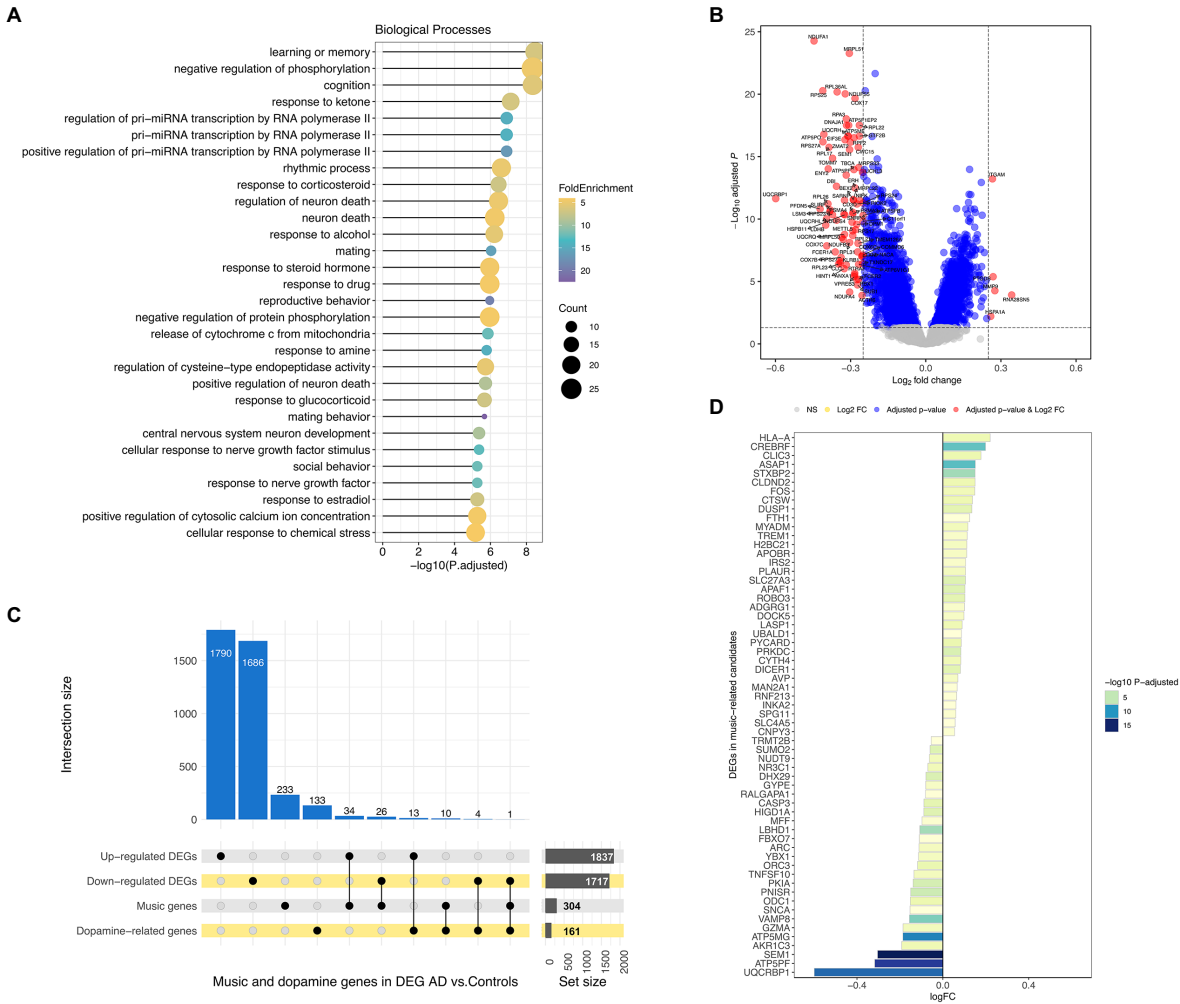
**(A)** Over-representation analysis of GO (Gene Ontology) terms using music-related candidate genes as input. Only the biological processes category was interrogated. **(B)** Volcano plot showing differentially expressed genes between AD patients and controls from the meta-analysis. **(C)** Upset plot of commonalities between up- and downregulated genes in AD patients, music-related and dopamine-related genes. **(D)** Differentially expressed genes in AD patients included in the music-related gene-set.

Among the biological processes in which the 334 music-related genes are involved, we found that learning / memory was the most significant pathway (*P*-adjusted = 3.3E-9). Other important processes related to cognition (*P*-adjusted = 4.4E-9), rhythm (*P*-adjusted = 2.5E-7) and neuron death regulation (*P*-adjusted = 2.3E-7) were present among the top 10 significant pathways (Supplementary Table S4; Figure 4A).

Some dopaminergic terms from GO were also significantly over-represented in the music-related gene-set, such as GO:0001963 (dopaminergic synaptic transmission, *P*-adjusted = 4.2E-5), GO:0042417 (dopamine metabolic process, *P*-adjusted = 0.002) or GO:0007212 (dopamine receptor signaling pathway, *P*-adjusted = 0.01). Interestingly, from the above list of the top six candidate genes, *SNCA* and *SLC6A4* are both involved in neuron–neuron synapse mediated by dopamine pathway (GO:0032227), as inferred by the protein–protein interaction network analysis.^2^ *SNCA* gene function is related to the regulation of dopamine release and transport, whereas *SLC6A4* is implicated in the regulation of serotonergic signaling by regulating serotonin reuptake into the presynaptic neuron, affecting emotions and stress responses.

### 4.2. Epigenomic commonalities between AD and music-related genes

There are 8,497 DMPs (FDR *p*-value <0.05) when contrasting AD patients vs. controls. 6,432 (75.7%) CpGs appeared as hypomethylated and 2,065 (24.3%) as hypermethylated in AD samples.

A total of 5,928 (69.8%) of these 8.4 k CpGs could be annotated to genes, resulting in 4,097 unique genes. Of the positions annotated to genes, 345 (4%) CpGs are annotated and related to more than one gene. For most of these genes, there are multiple DMPs associated (2.76 ± 1.28) gene-set and pathways enrichment analysis with the 8.4 k DMPs points to the following pathways depending on the database used: (a) KEGG: cytokine-cytokine receptor interaction, JAK/STAT signaling pathway, osteoclast differentiation, chemokine signaling pathway; (b) Reactome: neutrophil degradation, signaling by interleukins, nucleotide-binding domain, leucine rich repeat containing receptor (NLR) signaling pathways, platelet activation, signaling and aggregation, and (c) GO: leukocyte activation, cell activation, positive regulation of immune system process, immune effector process, cell migration, cell motility, localization of cell, immune response, locomotion. In addition, 853 DMRs (FDR *p*-value <0.05), containing at least three CpGs positions inside could be annotated to a total of 976 unique genes (only one DMR annotated to more than one gene). Of these regions, 645 (75.6%) appeared as hypomethylated in patients with AD, while the remaining 207 (24.3%) appeared as hypermethylated in cases vs. controls.

A total of 81 genes out of the 334 related to music (24.3%) were also found to be differentially methylated in the comparison of AD patients vs. HC, either considering genes associated with DMPs or those overlapping DMRs. Of these genes, 55 show an hypomethylation pattern in patients with AD when compared to HC, and 21 exhibit an opposite pattern of methylation; the remaining 5 genes, containing multiple CpGs inside, display both hypo- and hypermethylation patterns. The hypo and hypermethylation pattern, for most of these genes, resides within the gene body, while there are a small number of genes, such as *SLC6A4* and *AVPR1A*, where the aberrant methylation is observed within the promoter. The pathways analysis performed considering the 117 CpGs associated with the 81 music-related genes, revealed an enrichment for lysosome pathway in KEGG, for biological processes involved in nervous system development in GO, and for cadherin and Wnt signaling pathways, included in memory formation and synaptic plasticity and maintenance, in Panther.

In addition, 15 out of the 337 music-related genes (4.5%) match those associated with the DMRs that emerge when comparing AD patients and HC; 10 as hypermethylated in patients, and the remaining 5 as hypomethylated. The mean number of DMPs for these 15 genes was 3. The pathways analysis of the genes associated with DMRs reveal the significant enrichment of 356 unique GO terms, and 12 KEGG pathways. The enriched pathways in both categories include those related to T cell signaling pathways, myeloid lymphocyte differentiation, leukocyte mediated immunity, chemokine signaling pathways, etc.

### 4.3. Gene expression commonalities between Alzheimer’s disease and music-related genes

After data normalization and merging, a total of 19,145 genes were common when examining the three datasets of AD meta-analyzed in the present study. A quality control check of the normalized data through a principal component and clustering analysis highlighted 12 outlier samples belonging to the dataset GSE63061 that were finally disregarded for downstream analysis (GSM1539649, GSM1539651, GSM1539652, GSM1539653, GSM1539655, GSM1539644, GSM1539646, GSM1539647, GSM1539648, GSM1539650, GSM1539722, GSM4187601).

A total of 1,837 genes were found to be upregulated and 1,717 downregulated, all statistically significant in AD patients when compared against healthy controls (Figures 4B,C; Supplementary Table S5). The expression changes shown by DEGs are moderate but never too large [Log_2_FC ranging from −0.60 and 0.34; comparable to those reported in the literature (Wang et al., 2021; He et al., 2022)]; probably because the main physiopathological alterations in AD patients occur in the brain. The high number of DEGs detected, each with subtle gene expression alterations, therefore mirrors the pathological condition at systemic level.

304 out of the full list of 334 music-related candidate genes (91%) were also present in the AD meta-analysis. Notably, 60 of the 304 (20%) were among the DEGs observed when comparing AD patients and control samples (34 were upregulated and 26 downregulated; Figure 4D). Music-related genes showing the lowest *P*-adjusted value and higher log_2_FC were downregulated in AD vs. HC.

There is suggestive evidence indicating that dopaminergic pathways may play a role in the interplay between AD, memory, and music (see above). In our meta-analysis, when comparing transcriptomes from AD patients vs. healthy controls, the expression analysis showed a few DEGs involved in dopaminergic pathways (13 upregulated and 4 downregulated in AD vs. HC). In addition, there are dopaminergic genes among those in the music gene-set (*n* = 10), with *SNCA* (Synuclein Alpha) being the gene that best represents the AD-dopamine-music connection (Figure 4C; Supplementary Table S3).

### 4.4. Consensus co-expression analysis between Alzheimer’s disease and music conditions

To further investigate possible molecular links between AD and music-related genes, we generated a consensus co-expression network using AD patients and the only transcriptomic dataset related to musical stimuli available, aiming at detecting common conserved co-expression modules.

After applying a variance gene filtering (see Methodology section), 14,358 and 15,685 genes were retained in AD and music datasets, respectively, from which 11,920 genes were shared between both datasets and therefore available for follow-up consensus analysis. A total of 84 out of the 303 music-related gene-set in the meta-analysis (27.7%) did not pass the variance filtering; therefore, a reduced subset of 219 (72.3%) music-related genes was among the 11,920 genes used as input.

**Figure 5:**
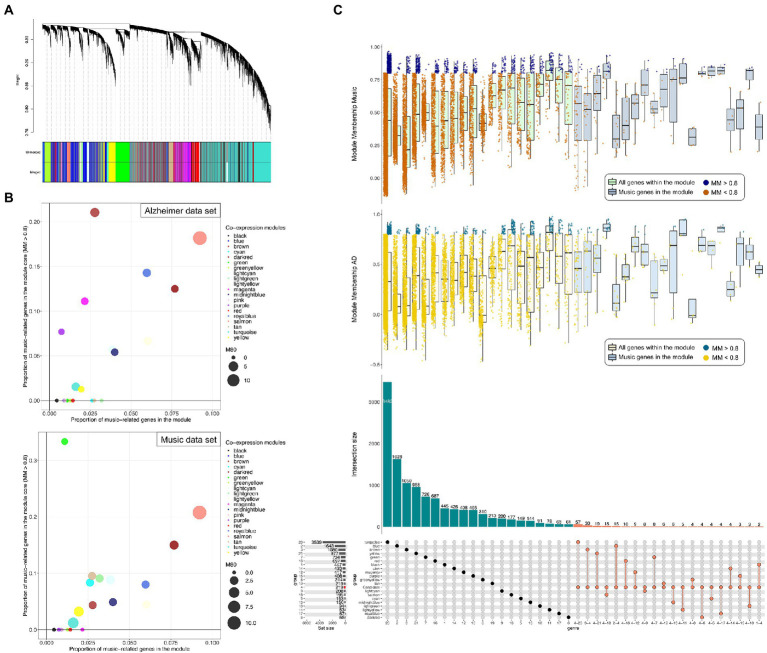
**(A)** Clustering dendrogram of genes and co-expression consensus modules detected, represented by different colors. **(B)** Scatter plots representing the proportion of music-related genes in the module core (Module membership [MM] > 0.8) against the proportion of music-related genes in the module. Size of the points is proportional to the number of genes in the core of the module (MM > 0.8). **(D)** Upset plot showing the proportion of music-related candidate genes included in the consensus modules (bottom). Boxplots (top) representing the MM value of the genes from each module from both the AD and music dataset, and horizontally separated in those included and not included in the music-related gene-set.

The consensus co-expression analysis revealed 20 co-expressed gene modules of very different sizes (ranging from 61 genes of the ‘darkred’ module, to 3,482 of the turquoise module; Figure 5A; Supplementary Figure S4A,B). The overall preservation of the eigengene networks was moderately high (*D* = 0.8; Supplementary Figure S4C) and the inter-module relationships in the two data sets has similarities. The highest eigengene preservation measurement was in the salmon and yellow modules (Supplementary Figure S4C).

The module with more music-related genes relative to the size of the module was the salmon one (18/195; Figure 5B); interestingly, most of them belong to the core of the module (11 genes in music dataset and 14 in the AD dataset with MM > 0.8; Figure 5C) suggesting a major role within it. Among the top 5 most connected genes within the module, we found 3 music-related genes: *GMPR* (top hub gene in both datasets with MM values of 0.95 and 0.93 in AD and music datasets respectively), *SELENBP1* and *ADIPOR1* (Supplementary Table S6).

## 5. Discussion

The prospect of slowing down of dementia onset and progression using music stimuli may seem utopian. Yet, a growing body of cognitive, neuroscience and genetic research is illuminating the possible benefits of music on cognition in AD patients. The study of musical sensogenomics in AD and other neurodegenerative diseases could help connect different fields of research to gain an integrated and interactive approach in a near future.

From a neuroscience perspective, music constitutes a powerful stimulus, specially for autobiographical memory, and many studies have emphasized the beneficial effect of music on the preservation of musical semantic memory, with special agreement on the ability to play a musical instrument (musical procedural memory). Since 1980, many studies have demonstrated the beneficial effect of musical stimuli on rehabilitation or improvement of AD patients, for self-consciousness, awareness, memory enhancement, cognitive function, or mood, among others, becoming a fruitful research field as a non-pharmacological intervention for neurodegenerative diseases. In this review, we specially analyze studies from 2000 to 2022, corroborating the impact of music in memory and cognition in AD as a powerful stimulus and as a therapy.

Many studies have highlighted the benefits of music in AD prevention and treatment, but molecular mechanisms underlying these observations have not been described (Baird et al., 2017; Innes et al., 2018; Moreira et al., 2018). Musical practice, and probably also music listening, has beneficial effects on the cognitive function and aging, increasing the brain plasticity and providing certain degree of neuroprotection (Balbag et al., 2014; Roman-Caballero et al., 2018).

We have detected a significant number of genes that have been previously reported to be related to music that are differentially expressed when comparing AD and HC. Three genes are particularly remarkable in the module gene expression analysis, namely, *GMPR*, *SELENBP1* and *ADIPOR1*. It is remarkable that these genes were previously related to neuropsychiatric and neurodegenerative diseases and, despite the limited number of molecular studies carried in music, a role has been reported for these genes in music performance (Kanduri et al., 2015a). The *GMPR* gene encodes for the human guanosine monophosphate reductase 1; it has been reported to show a gradual over-expression that increases with AD progression (Liu et al., 2018). This gene is involved in purine metabolism and recent data suggest that, altering the tight regulation of purine and pyrimidine metabolism may cause neuronal dysfunction, facilitating the onset of severe mental pathologies (Gottle et al., 2013; Fumagalli et al., 2017). *SELENBP1* has been found to be related with schizophrenia (Amar et al., 2010; Udawela et al., 2015; Chau et al., 2018) and was reported as differentially expressed in blood and brain samples of schizophrenia patients (Glatt et al., 2005). *ADIPOR1* encodes for the adiponectin receptor 1; it has beneficial effects on brain metabolism *via* AMP-activated protein kinase (AMPK) (Kim et al., 2017; Waragai et al., 2017); this gene was pointed out as a protection candidate against neuronal cell death and learning memory impairment, and is emerging as a potential therapeutic target in AD (Kim et al., 2017; Shah et al., 2017).

Dopamine is a neuromodulator of CNS released from the brain; it regulates several functions such as motor control, motivation, reward, cognitive function, learning, memory processing, and reproductive behaviors. Dopaminergic pathways have been recently connected with music stimulation processes, probably through reward mechanisms (Zatorre, 2015; Peck et al., 2016; Järvelä, 2018). In addition, the potential involvement of these signaling pathways in the onset and progression of some neurological disorders such as Parkinson’s (Cheng et al., 2010), schizophrenia (Owen et al., 2016), attention deficit and hyperactivity (Castellanos and Tannock, 2002; Gizer et al., 2009) and addiction (Baik, 2013) has been also pointed out. In line with these neuroscientific observations, our gene expression data suggest that there are connections between music, AD, and dopamine, represented by some common genes participating in these processes. One of the most remarkable genes involved in dopaminergic pathways is *SCNA*; a gene that also showed up as downregulated in the differential expression analysis of the AD dataset, and which is also included in the music-related gene-set (Supplementary Table S2). *SNCA* is mainly expressed in the brain, and it plays a role in the synaptic transmission. It has been associated with learning and memory, and neurodegenerative disorders such as Parkinson’s disease (Polymeropoulos et al., 1997; Kruger et al., 1998; Siddiqui et al., 2016) and AD (Hashimoto and Masliah, 1999; Twohig and Nielsen, 2019). The Synuclein Alpha encoded by this gene is one of the major components of Lewy bodies in PD, and in the amyloid plaques located in the brain of AD patients.

Of the 334 genes associated with music, 127 appeared as linked with epigenome/transcriptome modules regarding AD. Six of these highlighted genes in the ‘omic’ analysis are particularly interesting, namely, *SNCA*, *SLC6A4*, *ASCC2*, *FTH1*, *PLAUR*, *ARHGAP26*, because they have been previously associated with musical aptitude, music effects on transcriptional activity, or AD (Supplementary Table S2). It is remarkable that, although many of the studies carried out on biomolecular markers and musical conditions are limited in sample size, a few genes appear repeatedly in both music related studies and research on neurodegenerative disorders.

Several concerns have been raised regarding studies conducted on music and memory. Baird and Samson (2015) indicate that “The recent findings that musical training delays cognitive decline and promotes plasticity in the elderly brain are promising. There is an urgent need for further methodologically rigorous investigations of this topic in light of our rapidly aging population and the corresponding increasing incidence of dementia.” Several studies have also questioned the mechanisms involved in the effectiveness of music therapy, but eluded genomics (Clements-Cortes and Bartel, 2018). Further authors have lamented the scarcity of rigorous scientific investigation of music cognition in dementia (Samson et al., 2012; Baird and Samson, 2015). In the same vein, Cohen-Mansfield (2014) noted the poor utilization of the literature and low ecological validity. Moreover, recent randomized control studies have questioned the effect of music in AD patients, e.g., (Kwak et al., 2020).

There are several limitations in the present study. On the neuroscientific articles systematically reviewed, we noticed that the methodology employed in the literature is highly heterogeneous as well as the measurements used to evaluate the beneficial effects of music. The lack of a consensus and standardized methodology in these studies limits the possibility to meta-analyze the findings. More rigorous clinical trials are still needed (Bian et al., 2021), as well as the development of standardized research protocols allowing to evaluate the improvements provided by the musical stimuli (Yin et al., 2022) in a homogenous way. Despite these limitations, a close inspection of the specialized literature unambiguously indicate that music has a beneficial impact on health, being memory and cognition the most common outcomes. On the ‘omic’ side, the search for common gene expression patterns and epigenomics between AD and music are also confronted with many limitations, which inevitably limit the scope of our study. The most important limitation comes from the very scarce number of studies available on gene expression (and none on epigenomics) regarding music. To overcome this limitation, we used here a novel approach that takes advantage of crossmatching the best collection of musical gene candidates (as previously reported in the literature on genomics and transcriptomics) with transcriptomic and epigenomics publicly available data. This approach has allowed to reveal new features that are particularly relevant to AD, as it is the fact that there are many genes related to music that are also differentially expressed in AD patients (when compared to controls). Understanding the meaning of this overlap will require new experimental designs aimed at investigating the effects of the musical stimuli on the transcriptomes and epigenomes, in line with the aims pursued by musical sensogenomics (Navarro et al., 2021). Exploring molecular links between AD and music stimuli could help to illuminate new therapeutical targets.

In the present study, neurosciences, cognitive sciences, epigenetics, transcriptomics, and genetics were brought together in the search for connections between music and memory. Our findings represent a molecular proof of concept that establishes a genetic link between music and AD; however, further effort is needed to understand this gene commonality. As an initial step, the analysis of the transcriptome response to music stimuli using controlled experimental designs and cohorts are mandatory.

## Data availability statement

Publicly available datasets were analyzed in this study. This data can be found here: Gene Expression Omnibus (GEO) database: GSE140829, GSE63061, GSE63060, GSE97760, and GSE153712.

## Author contributions

AS, FM-T, and LN conceived the study. LN and ACM carried out a systematic review of the literature on neuroscience and genomics. AG-C, SP, JM-L, SV-L, and AS contributed to the analysis of the data. AS, LN, AG-C, and SP wrote the first draft of the paper and the rest of the authors made contributions to it. All the authors approved the final version of the manuscript.

## Conflict of interest

The authors declare that the research was conducted in the absence of any commercial or financial relationships that could be construed as a potential conflict of interest.

## Publisher’s note

All claims expressed in this article are solely those of the authors and do not necessarily represent those of their affiliated organizations, or those of the publisher, the editors and the reviewers. Any product that may be evaluated in this article, or claim that may be made by its manufacturer, is not guaranteed or endorsed by the publisher.

## Footnotes

^1^ https://doi.org/10.1093/nar/gkz393

^2^ www.string-db.org
