# Supplementary material for: Sensogenomics of music and Alzheimer’s disease: An interdisciplinary view from neuroscience, transcriptomics, and epigenomics
Source: Front Aging Neurosci. 2023 Feb 3;15:1063536. doi: 10.3389/fnagi.2023.1063536 (PMC9935844; doi:10.3389/fnagi.2023.1063536)
Supplement: Supplementary file 1 [file Data_Sheet_1.zip › Text.DOCX]

Supplementary Text

**Sensogenomics of music and Alzheimer’s disease: An interdisciplinary view from neuroscience, transcriptomics, and epigenomics**

Laura Navarro, Alberto Gómez-Carballa, Sara Pischedda, Julián Montoto-Louzao, Sandra Viz-Lasheras, Alba Camino-Mera^1,2,3^, Thomas Hinault, Federico Martinón-Torres, Antonio Salas

**Index**

1. Neural correlates of music in Alzheimer’s disease

2. Dopaminergic pathways connecting music and memory in Alzheimer disease

3. References

**1. Neural correlates of music in Alzheimer’s disease**

Listening to music involves different brain substrates and many cognitive elements [1]. It has been hypothesized that identification of familiar music and discrimination of musical elements involve different neurological mechanisms. Neuroimaging and lesion studies have tried to identify brain areas involved in musical memory function when listening to familiar music. Janata [2] discovered the implication of dorsal medial prefrontal cortex in autobiographical musical memory. Other studies focused on the recognition or imagery of familiar melodies have identified the activation of the temporal lobe regions and superior temporal gyrus [3], the right temporal pole [4], frontal regions and middle frontal lobes [5; 6], or a distributed network of perisylvian areas [1; 7]. Johnson et al. [8] found that temporal lobes play an important role in the recognition of familiar music, but also that different forms of dementia respond differentially to music. In line with previous functional imaging studies, Platel et al. [9] have investigated the different neural substrates underlying the semantic and episodic memory, discovering that familiar and nonfamiliar melodic tunes showed distinct neural networks. In contrast, by employing unrecognized music based on newly composed materials, Watanabe et al. [10], discovered that the right hippocampus responded to the retrieval success more strongly than the left hippocampus, and the left inferior frontal gyrus (IFG) responded more strongly than the right IFG.

**2. Dopaminergic pathways connecting music and memory in Alzheimer disease**

A large body of evidence in neuroscience has established the role of the dopamine system in processing reward in connection with music and/or memory. Peck et al. [11] established three mechanisms that can be hypothesized to underlie the beneficial effect that music plays in enhancing memory with AD: (*i*) the activation of dopaminergic pathways, (*ii*) the activation of the autonomic nervous system, specifically the sympathetic nervous system, and (*iii*) the default neuronal connectivity. These authors have claimed that “music can be used to stimulate and strengthen dopaminergic pathways and interconnected brain structures that are commonly compromised in persons with Alzheimer’s disease”.

Based on pioneering research, Menon and Levitin (2005) claimed that key, unreported importance of the ventral tegmental area (VTA) and the nucleus accumbens (NAcc) in music processing. Music accesses and engages brain systems associated with reward and emotion, activating the dopaminergic system [12; 13; 14; 15; 16]. However, AD patients have an impaired dopaminergic system, because they suffer dysfunctions from the VTA to the NAcc [17]. Dopaminergic depletion has also been documented in persons with Parkinson’s disease e.g., [18; 19]. Dopaminergic cell bodies are located in the VTA, considered the starting point of two dopaminergic pathways: mesolimbic and mesocortical [20]. Also, the VTA projects to other cortical areas involved in memory consolidation [21]. Furthermore, according to Wise [22] dopamine release is implicated in the reinforcement of long-term memory (LTM), motivation and motor function. Moreover, AD patients suffer from degenerative changes in substantia nigra, responsible for dopamine generation [17].

The affection of the mesolimbic pathway has serious consequences in AD patients because it plays a fundamental role in the reward system, connecting the VTA to the ventral striatum to release the dopamine [23; 24]. According to Blum et al. [25], the limbic system is the core region of reward pathway, and music listening activates mesolimbic experiences. Other studies support this idea with remarkable findings [12; 14; 15; 26; 27]. For example, Salimpoor et al. [14] confirmed that intense emotional responses to music trigger the dopamine release in the striatum (ventral and dorsal), especially when music is pleasurable and familiar. However, when music is pleasurable but new, only the NAcc (in the ventral striatum), considered “the most important pleasure center” (Marvidis, 2015) and auditory cortices are connected [15]. In fact, a possible explanation of musical anhedonia is the lack of interaction between the striatum and the auditory mechanism [28], or reduced activation in the brain structures involved in reward (NAcc and VTA, among others) [29].

**References**

[1] L. Stewart, K. von Kriegstein, J.D. Warren, and T.D. Griffiths, Music and the brain: disorders of musical listening. Brain : a journal of neurology 129 (2006) 2533-53.

[2] P. Janata, The neural architecture of music-evoked autobiographical memories. Cereb Cortex 19 (2009) 2579-94.

[3] M. Satoh, K. Takeda, K. Nagata, E. Shimosegawa, and S. Kuzuhara, Positron-emission tomography of brain regions activated by recognition of familiar music. AJNR. American journal of neuroradiology 27 (2006) 1101-6.

[4] S. Hsieh, M. Hornberger, O. Piguet, and J.R. Hodges, Neural basis of music knowledge: evidence from the dementias. Brain 134 (2011) 2523-34.

[5] R.J. Zatorre, A.R. Halpern, D.W. Perry, E. Meyer, and A.C. Evans, Hearing in the Mind's Ear: A PET Investigation of Musical Imagery and Perception. J Cogn Neurosci 8 (1996) 29-46.

[6] A.R. Halpern, and R.J. Zatorre, When that tune runs through your head: a PET investigation of auditory imagery for familiar melodies. Cereb Cortex 9 (1999) 697-704.

[7] H. Platel, J.C. Baron, B. Desgranges, F. Bernard, and F. Eustache, Semantic and episodic memory of music are subserved by distinct neural networks. Neuroimage 20 (2003) 244-56.

[8] J.K. Johnson, C.C. Chang, S.M. Brambati, R. Migliaccio, M.L. Gorno-Tempini, B.L. Miller, and P. Janata, Music recognition in frontotemporal lobar degeneration and Alzheimer disease. Cogn Behav Neurol 24 (2011) 74-84.

[9] H. Platel, Functional neuroimaging of semantic and episodic musical memory. Ann N Y Acad Sci 1060 (2005) 136-47.

[10] T. Watanabe, S. Yagishita, and H. Kikyo, Memory of music: roles of right hippocampus and left inferior frontal gyrus. Neuroimage 39 (2008) 483-91.

[11] K.J. Peck, T.A. Girard, F.A. Russo, and A.J. Fiocco, Music and Memory in Alzheimer's Disease and The Potential Underlying Mechanisms. J Alzheimers Dis 51 (2016) 949-59.

[12] A.J. Blood, and R.J. Zatorre, Intensely pleasurable responses to music correlate with activity in brain regions implicated in reward and emotion. Proc Natl Acad Sci U S A 98 (2001) 11818-23.

[13] V.N. Salimpoor, M. Benovoy, G. Longo, J.R. Cooperstock, and R.J. Zatorre, The rewarding aspects of music listening are related to degree of emotional arousal. PLoS One 4 (2009) e7487.

[14] V.N. Salimpoor, M. Benovoy, K. Larcher, A. Dagher, and R.J. Zatorre, Anatomically distinct dopamine release during anticipation and experience of peak emotion to music. Nat Neurosci 14 (2011) 257-62.

[15] V.N. Salimpoor, I. van den Bosch, N. Kovacevic, A.R. McIntosh, A. Dagher, and R.J. Zatorre, Interactions between the nucleus accumbens and auditory cortices predict music reward value. Science 340 (2013) 216-9.

[16] D. Sutoo, and K. Akiyama, Music improves dopaminergic neurotransmission: demonstration based on the effect of music on blood pressure regulation. Brain research 1016 (2004) 255-62.

[17] W.R. Gibb, C.Q. Mountjoy, D.M. Mann, and A.J. Lees, The substantia nigra and ventral tegmental area in Alzheimer's disease and Down's syndrome. Journal of neurology, neurosurgery, and psychiatry 52 (1989) 193-200.

[18] J.M. Shine, P.T. Bell, E. Matar, R.A. Poldrack, S.J.G. Lewis, G.M. Halliday, and C. O'Callaghan, Dopamine depletion alters macroscopic network dynamics in Parkinson's disease. Brain : a journal of neurology 142 (2019) 1024-1034.

[19] S.J. Chung, H.S. Lee, H.S. Yoo, Y.H. Lee, P.H. Lee, and Y.H. Sohn, Patterns of striatal dopamine depletion in early Parkinson disease: Prognostic relevance. Neurology 95 (2020) e280-e290.

[20] K.T. Demarest, and K.E. Moore, Comparison of dopamine synthesis regulation in the terminals of nigrostriatal, mesolimbic, tuberoinfundibular and tuberohypophyseal neurons. J Neural Transm 46 (1979) 263-77.

[21] J. Posner, J.A. Russell, and B.S. Peterson, The circumplex model of affect: an integrative approach to affective neuroscience, cognitive development, and psychopathology. Dev Psychopathol 17 (2005) 715-34.

[22] R.A. Wise, Dopamine, learning and motivation. Nat Rev Neurosci 5 (2004) 483-94.

[23] V. Menon, and D.J. Levitin, The rewards of music listening: response and physiological connectivity of the mesolimbic system. Neuroimage 28 (2005) 175-84.

[24] K.C. Berridge, and T.E. Robinson, What is the role of dopamine in reward: hedonic impact, reward learning, or incentive salience? Brain Res Brain Res Rev 28 (1998) 309-69.

[25] K. Blum, T.J. Chen, A.L. Chen, M. Madigan, B.W. Downs, R.L. Waite, E.R. Braverman, M. Kerner, A. Bowirrat, J. Giordano, H. Henshaw, and M.S. Gold, Do dopaminergic gene polymorphisms affect mesolimbic reward activation of music listening response? Therapeutic impact on Reward Deficiency Syndrome (RDS). Med Hypotheses 74 (2010) 513-20.

[26] R.J. Zatorre, and V.N. Salimpoor, From perception to pleasure: music and its neural substrates. Proc Natl Acad Sci U S A 110 Suppl 2 (2013) 10430-7.

[27] I.N. Mavridis, Music and the nucleus accumbens. Surgical and radiologic anatomy : SRA 37 (2015) 121-5.

[28] R.J. Zatorre, Musical pleasure and reward: mechanisms and dysfunction. Ann N Y Acad Sci 1337 (2015) 202-11.

[29] J. Keller, C.B. Young, E. Kelley, K. Prater, D.J. Levitin, and V. Menon, Trait anhedonia is associated with reduced reactivity and connectivity of mesolimbic and paralimbic reward pathways. J Psychiatr Res 47 (2013) 1319-28.
