# Supplementary figures and images for: Sensogenomics of music and Alzheimer’s disease: An interdisciplinary view from neuroscience, transcriptomics, and epigenomics
Source: Front Aging Neurosci. 2023 Feb 3;15:1063536. doi: 10.3389/fnagi.2023.1063536 (PMC9935844; doi:10.3389/fnagi.2023.1063536)

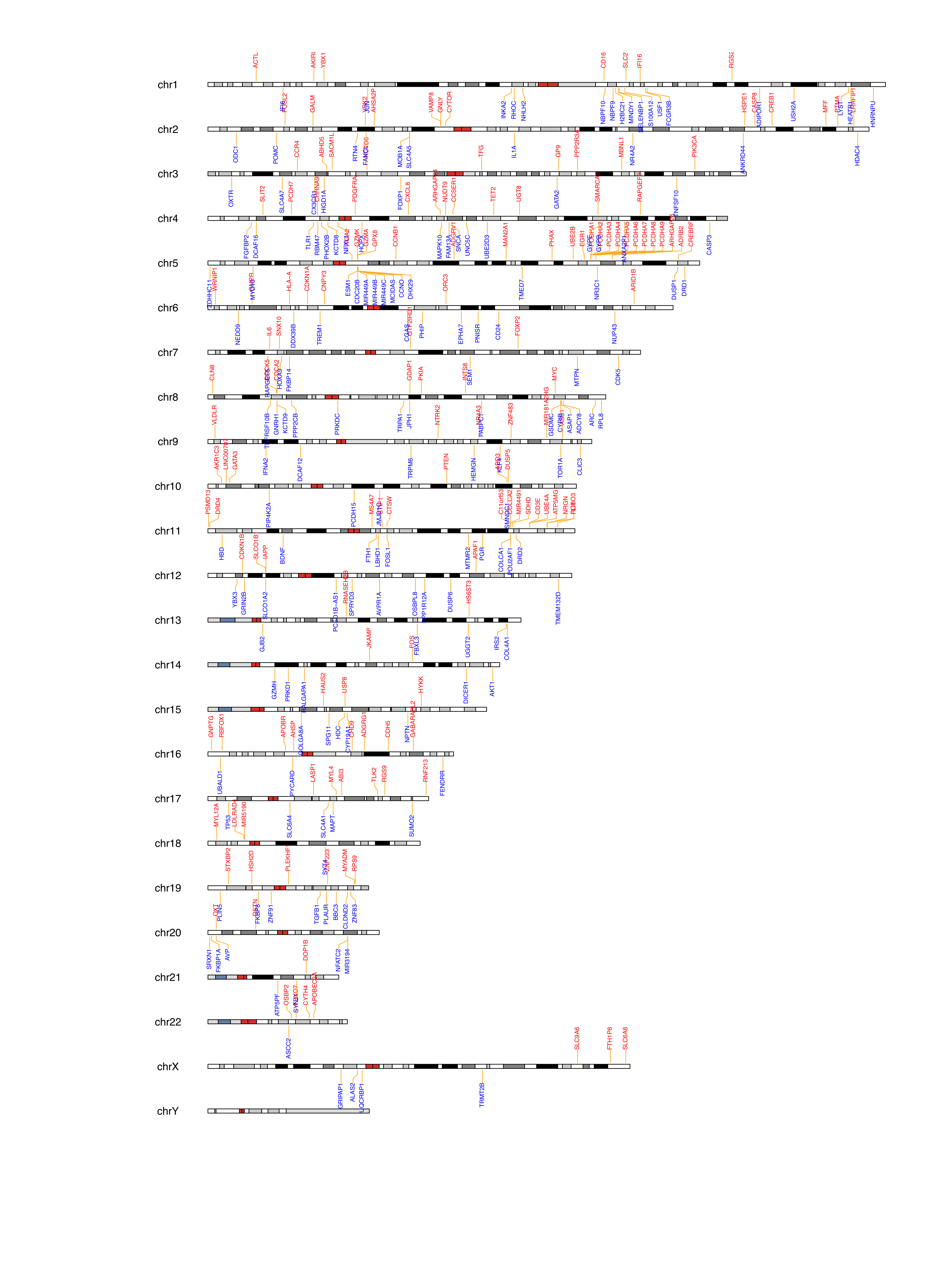

Supplement: Supplementary file 1 [file Data_Sheet_1.zip › Image 1.TIF]

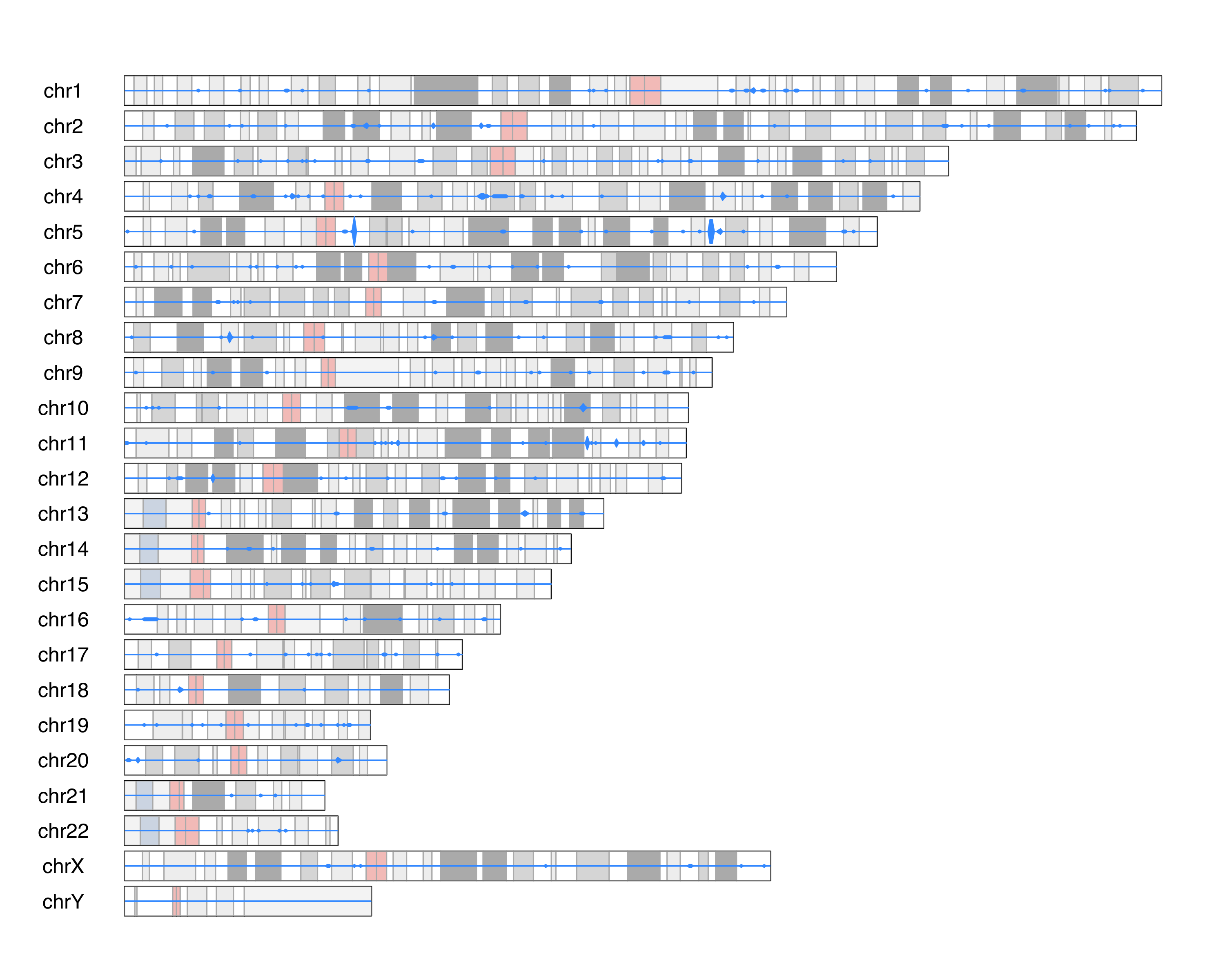

Supplement: Supplementary file 1 [file Data_Sheet_1.zip › Image 2.TIF]

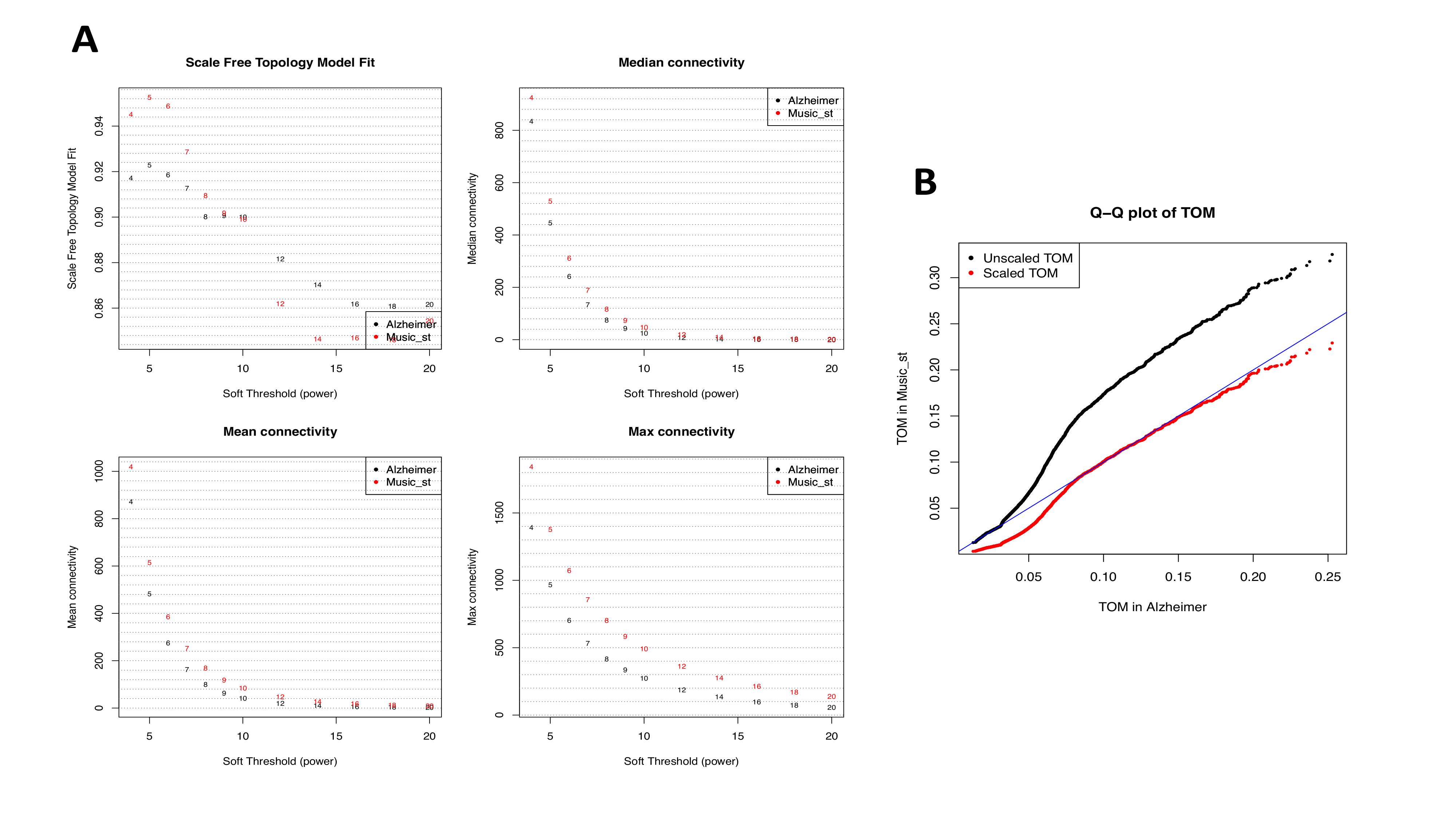

Supplement: Supplementary file 1 [file Data_Sheet_1.zip › Image 3.TIF]

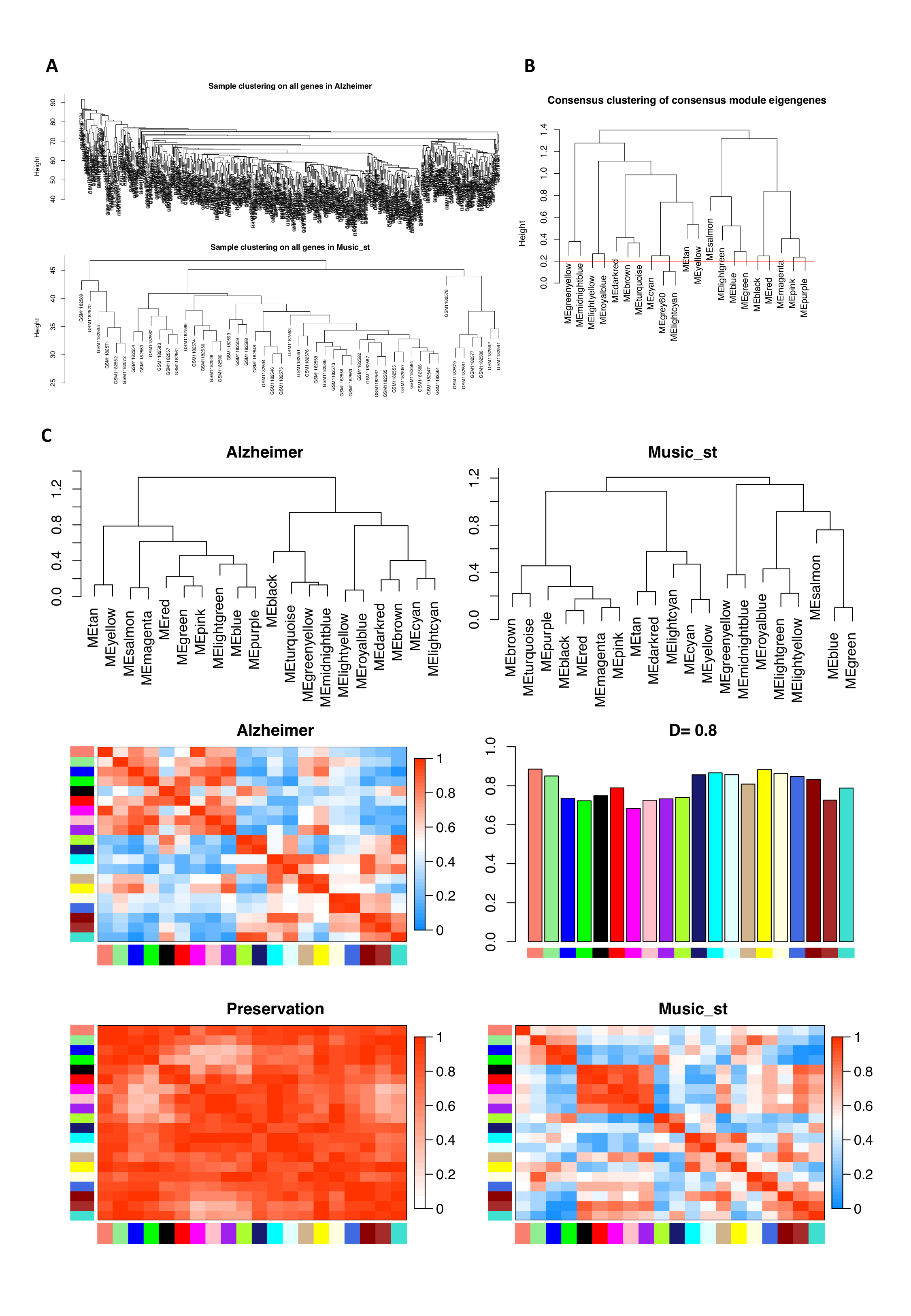

Supplement: Supplementary file 1 [file Data_Sheet_1.zip › Image 4.TIF]
